# Evaluating a Living Donor With Rheumatoid Arthritis for a Recipient With End-Stage Renal Disease From Antineutrophil Cytoplasmic Antibodies Associated Vasculitis

**DOI:** 10.7759/cureus.18117

**Published:** 2021-09-20

**Authors:** Luisa M De Souza, Nasrollah Ghahramani, Catherine Abendroth, Gurwant Kaur

**Affiliations:** 1 Medicine, Penn State University College of Medicine, Milton S. Hershey Medical Center, Hershey, USA; 2 Nephrology, Penn State Health Milton S. Hershey Medical Center, Hershey, USA; 3 Pathology, Penn State Health Milton S. Hershey Medical Center, Hershey, USA

**Keywords:** anca-associated vasculitis, esrd, living kidney donor, rheumatoid arthritis, kidney transplantation

## Abstract

A 60-year-old Caucasian female with sero-positive rheumatoid arthritis (RA) was evaluated as a potential kidney donor for her brother-in-law with end-stage kidney disease (ESKD) secondary to c-antineutrophil cytoplasmic antibody (c-ANCA) associated vasculitis (AAV) and membranous nephropathy (MN). With little to no data supporting or contradicting this unique scenario, in addition to the varying viewpoints expressed by the different specialists, our multidisciplinary transplant committee encountered a difficult decision of whether to approve a candidate with RA for a living kidney donation or not. As a result, we carried out a careful literature review addressing aspects of recipients’ outcomes following kidney transplants from a living donor with RA, especially when the recipient has AAV, living donor’s short- and long-term outcomes post kidney donation, renal disease in AAV and RA, and maintenance of disease remission.

## Introduction

A 60-year-old Caucasian female with sero-positive rheumatoid arthritis (RA) was evaluated as a potential kidney donor for her brother-in-law with end-stage kidney disease (ESKD) secondary to c- antineutrophil cytoplasmic antibody (c-ANCA) associated vasculitis (AAV) and membranous nephropathy (MN). It is widely accepted that patients with RA are likely to experience a decline in kidney function over time [1]. The direct effects of RA on the kidney, although rare, include the potential development of mesangial proliferative glomerulonephritis (GN), amyloidosis, focal proliferative GN, membranous GN, among others [2-4]. Drug toxicity of anti-rheumatological agents, such as nonsteroidal anti-inflammatory drugs (NSAIDs) and cyclosporine, are the major contributors to RA-associated renal disease [3]. For this reason, the risk of renal failure in living kidney donors with RA remains an ongoing concern. Fortunately, a handful of studies have shown positive post-surgical outcomes in this patient population. A retrospective cohort study demonstrated that living kidney donors suffering from RA, who are otherwise healthy, experience good outcomes in the long term [5].

Active systemic diseases such as ANCA-AAV might be considered a relative contraindication for receiving kidney transplantation due to the risk of disease recurrence in the early post-transplant period. However, appropriate graft function and post-transplant patient survival have been widely reported in such scenarios [6-15]. For this reason, despite the recurring nature of this disease, renal transplantation should be offered once they are eligible for patients with ESKD secondary to AAV. At the same time, the literature detailing the recurrence of AAV among kidney allograft recipients is equally extensive and compelling [16-33].

## Case presentation

Patient vignette: the recipient

**Figure 1:**
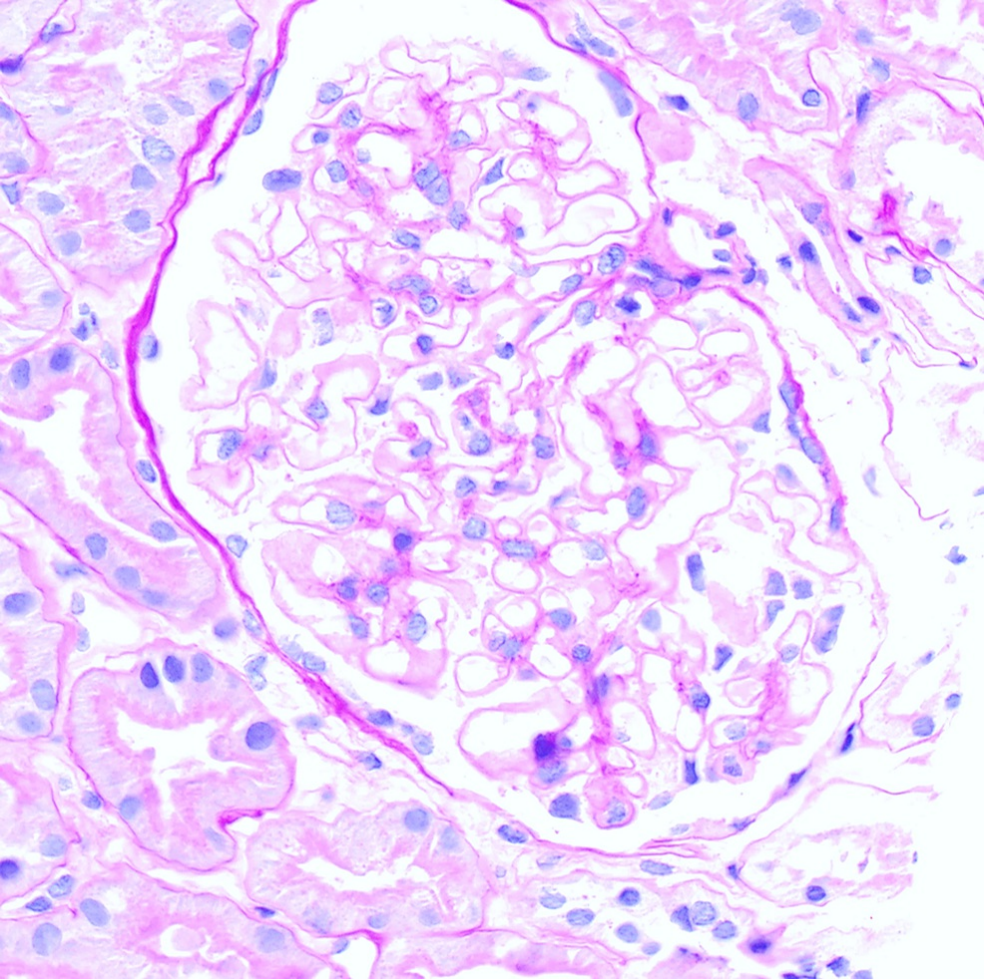
Normocellular glomerulus; capillary walls are normal thickness (PAS x500).

**Figure 2:**
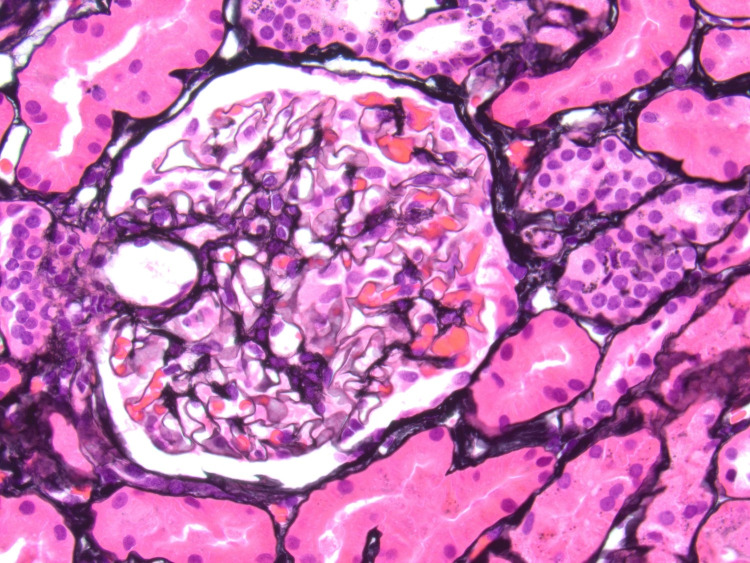
No basement membrane spikes visible on silver staining (Jones x500).

**Figure 3:**
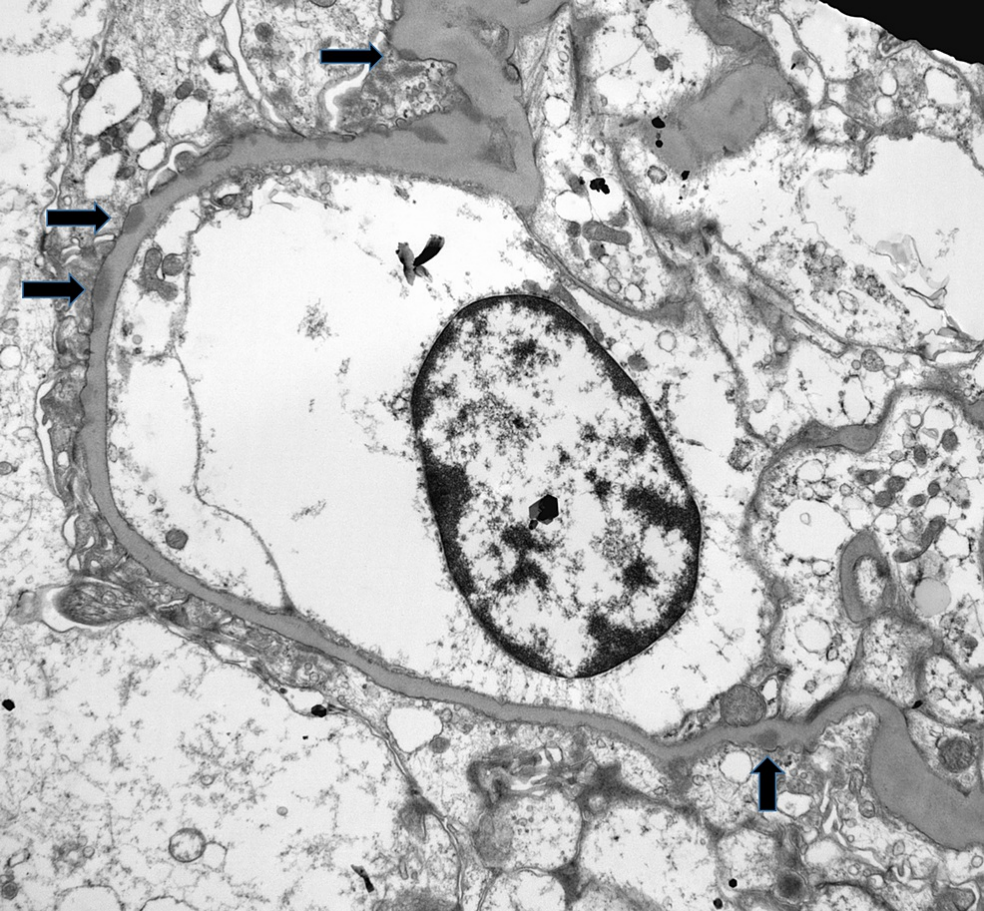
Small subepithelial electron-dense deposits revealed by electron microscopy (arrows). Minimal to absent new basement membrane material, no clear “spikes” (original magnification x5,800).

A 62-year-old Caucasian male with ESKD was evaluated in a pre-transplant Nephrology clinic in 2019. Pertinent past history included the discovery of sub-nephrotic proteinuria (720 mg/day) in 2013 while applying for life insurance. Kidney biopsy demonstrated minimal alterations at the light microscopic level (Figures 1, 2). Immunofluorescence studies revealed finely granular immunoglobulin G (IgG) dominant capillary wall immunoreactivity. Electron microscopy demonstrated very small subepithelial electron-dense deposits consistent with early-stage membranous nephropathy (MN) (Figure 3). Antinuclear antibodies (ANAs), hepatitis panel, complement levels, human immunodeficiency virus (HIV), and immunofixation were all negative or within normal limits at the time of biopsy. In June of 2018, he presented to an outside hospital (OSH) with complaints of dyspnea, cough, and hemoptysis, and was found to have a large left pleural effusion. Thoracocentesis revealed an exudative pattern with negative cytology. He was treated conservatively for community-acquired pneumonia (CAP).

On the first readmission, he had edema and pleural, and pericardial effusions. He was managed with therapeutic thoracocentesis, pericardiocentesis, and pericardial drain. Pleural and pericardial fluid (hemorrhagic) studies revealed no malignant cells. Bronchoscopy ruled out any possible endobronchial malignancy. At this point, the patient was found to be c-ANA positive (1:320 titter) with an elevated IgG class ANCA directed to proteinase 3 (PR3) at 437.4, rheumatoid factor (RF) at 255 IU/mL, C-reactive protein (CRP) at 3.2 mg/dL, and IgA beta-2 glycoprotein at 63 Standard IgG units, confirming a diagnosis of vasculitis. ANA, perinuclear anti-neutrophil cytoplasmic antibodies (p-ANCA), anticardiolipin antibodies, and beta-2 glycoprotein IgG and immunoglobulin M (IgM) were within normal limits. A second native kidney biopsy was performed at this time due to worsening renal function. It showed possible acute tubular necrosis (ATN) superimposed on background MN. His abdominal imaging revealed splenic and renal infarcts as well. We were unable to get pathology pictures due to his care being at an OSH.

His second readmission was significant for acute hypoxic respiratory failure due to pleural and pericardial effusions, and definitive management was achieved via pericardial window placement and left chest decortication. Surgical pathology revealed necrotizing fibrinoid changes within lung tissue along with vascular necrosis, highly suggestive of small vessel disease with granulomatosis with polyangiitis (GPA) (formerly known as Wegener’s granulomatosis). Interventional radiology (IR) arteriogram demonstrated an irregularly beaded morphology of the renal, hepatic, splenic, cystic, pancreaticoduodenal, jejunal, and colic arteries concerning medium vessel disease such as Polyarteritis Nodosa vs. atypical AAV. Eventually, he required renal replacement therapy likely due to advanced MN, initially via intermittent hemodialysis in August of 2018, and later was transitioned to peritoneal dialysis (PD) in May of 2019. He was assessed at Hershey Medical Center (HMC) for pre-transplant evaluation in August of 2019, at which point he was deemed a suitable candidate.

Patient vignette: the donor

A 60-year-old Caucasian female sero-positive for both rheumatoid factor (RF) and anti-cyclic citrullinated peptide (CCP) positive RA was evaluated as a potential living kidney donor. She presented to the donor evaluation clinic with a desire to donate a kidney to her brother-in-law (above mentioned potential recipient). Her RA was first diagnosed 18 years ago when she presented to an outside clinic with complaints of symmetrical large and small joint tenderness with morning stiffness, primarily affecting the shoulders and knees. Her family history was significant for fibromyalgia in her mother, who passed away at the age of 78. Her medical history was significant for gastroesophageal reflux disease (GERD) and Barret’s esophagus secondary to peptic ulcer disease, as well as morbid obesity with body mass index (BMI) of 36 kg/m^2^ requiring gastric bypass surgery in 2013. Social history was significant for 15 pack-years of former smoking. During the early stages of her RA, she had briefly received methotrexate (MTX) and prednisone. She was later transitioned to hydroxychloroquine 200 mg twice daily, with significant symptom improvement, but devoid of clinical remission. NSAIDs were avoided due to her extensive gastrointestinal history.

Laboratory data from 2019 showed that her RF was 18 IU/mL, anti-CCP > 250 U/mL, erythrocyte sedimentation rate (ESR) 6 mm/h, and CRP 2.3 mg/L. No tests for ANA, Smith antibodies (anti-Sm), ribonucleoprotein (RNP), topoisomerase, Sjögren's-syndrome-related antigen A (SSA), Sjögren's-syndrome-related antigen B (SSB), or double-stranded deoxyribonucleic acid (ds-DNA) antigens were found on her outside records.

At the time of living kidney donor assessment, the patient was still experiencing prolonged morning stiffness, as well as occasional pain flares in her hands and knees, mitigated by low-dose Tylenol. Complete blood count (CBC) was unremarkable, except for signs of possible reactive polycythemia, likely secondary to obesity hypoventilation syndrome (Table 1). Her serum creatinine was at 0.72 mg/dL, estimated glomerular filtration rate (eGFR) >60 mL/min/1.73 m^2^, and no indication of Type II Diabetes Mellitus. Urinalysis showed an elevated leukocyte esterase, proteinuria and microscopic hematuria, and pyuria, without evidence of urinary tract infection (UTI). Her candidacy as a living kidney donor posed a unique clinical scenario, as the potential recipient had a history of ESKD secondary to atypical c-ANCA-AAV and MN. Given the paucity of evidence supporting this unique clinical scenario, the institution’s transplant committee expressed concern for the uncertainty in both the donor’s and the recipient’s post-surgical outcomes.

**Table 1:** Potential kidney donor laboratory data NA denotes not available, blue denotes low levels, while red denotes high levels, φ Reference values are affected by many variables not limited to patient population and laboratory methods used. The ranges used at Hershey Medical Center are for nonpregnant adults and do not have medical conditions that could affect the results. They may therefore not be appropriate for all patients.

Variable	Reference Range, Adults φ	Pre-Transplant Evaluation
Blood		
Hematocrit (%)	35-44	46.2
Hemoglobin (g/dlL)	11.7-15	14.4
White cells (per µL)	4000-10,400	6,930
Platelets (per µL)	150,000-350,000	240,000
Differential count (per µL)		
Neutrophils	2,000-7,700	6,990
Lymphocytes	1,000-3,400	2,020
Monocytes	0-1,000	710
Eosinophils	0-500	230
Prothrombin time (sec)	12.0-14.2	13.4
International normalized ratio (INR)	0.9-1.1	1.0
Sodium (mmol/liter)	136-145	142
Potassium (mmol/liter)	3.5-5.1	4.1
Chloride (mmol/liter)	98-107	103
Carbon dioxide (mmol/liter)	22-29	28
Anion gap (mmol/liter)	5-14	11
Urea nitrogen (mg/dL)	6-23	8
Creatinine (mg/dL)	0.60-1.00	0.72
Estimated GFR (mL/min/1.73 m^2^)	>60	>60
Glucose (mg/dL)	74-109	90
Urine		
Sample appearance	Yellow	Yellow, slightly cloudy
Glucose	Negative	Negative
Ketones	Negative	Negative
Bilirubin	Negative	Negative
Specific gravity	1.005-1.030	1.023
pH	5.0-8.0	5.0
Leukocyte esterase	Negative	Large
Nitriles	Negative	Negative
Bacteria	None	None
Protein (mg/dL)	None	30
White blood cells (per high-power field)	0-4	10-19
Red blood cells (per high-power field)	0-4	5-9
Creatinine (mg/dL)	Not established	179.82
Protein ran (mg/dL)	<25 mg/dL	15
Prot:creat ratio	<0.18	0.08

## Discussion

Disease process in AAV

AAV pertains to a group of systemic inflammatory diseases, the hallmark of which is the necrotizing inflammation of mostly small vessels. Small vessel diseases come in three different types: GPA, microscopic polyangiitis (MPA), and eosinophilic GPA (formerly Churg-Strauss disease). Both GPA and MPA are associated with the presence of circulating Anti-ANCA; a specific type of autoantibodies targeted against either proteinase 3 (PR3-ANCA, in the case of GPA) or myeloperoxidase (MPO-ANCA, in the case of MPA). In particular, GPA is characterized by necrotizing inflammatory involvement of the ears-nose-and-throat (ENT), the lungs, and the kidneys. As in the case of our potential recipient, atypical manifestations of GPA have also been reported [34]. Due to its regular involvement of kidney vasculature, renal disease is a common complication in patients with AAV, presenting as crescentic GN with the characteristic absence or paucity of glomerular immunoglobulin deposits (pauci-immune) [35]. The renal disease occurs in over 80% of patients with AAV [36]. Out of this cohort, 20%-40% eventually develop ESKD within a five year period, as either firsthand rapidly progressive GN (RPGN), chronic kidney disease (CKD), or like in the case of our potential transplant recipient, via hemodynamically-associated renal dysfunction causing the natural progression of this systemic disease [36-39].

The vasculitis group was once considered to be a serious life-threatening disease cohort. However, the introduction of modern immunosuppressive therapy, with a lower degree of toxicity, has marked the milestone of patient survival. Despite medical advances, patients’ quality of life remains severely poor. Similar to our recipient’s complex clinical vignette, patients frequently accrue significant damage, stemming from the vasculitis process itself (e.g., lung fibrosis, ESKD), side effects of administered treatment (e.g., infections, osteoporosis), and worsening of preexisting comorbidities (e.g., hypertension, obesity) [40]. In addition, the burden of immunosuppression is further augmented by the frequent relapses experienced as part of the disease’s natural course, a characteristic commonly observed in PR3-ANCA positive AAV [41]. AAV treatment in patients who have not reached ESKD is usually biphasic, with the goal of inducing remission (3-6 months) for tight initial disease control, followed by remission maintenance to prevent relapses. For patients whose eGFR reaches < 30 mL/min/1.73 m^2^, referral to transplantation is the next course of action [42]. At this point, time becomes a precious commodity. Even though this was of little relevance to our recipient, studies have reported that patient and graft survival is improved when the first transplant occurs before the start of renal placement therapy (pre-emptive) [43].

Outcomes of kidney transplantation in AAV

A meta-analysis conducted in Canada demonstrated that patients with AAV have a 2.7-fold increase in mortality compared with the general population [44]. However, as previously mentioned, multiple studies have shown that patients with AAV do fairly well following renal transplantation [6-15]. One cohort study, in particular, demonstrated that patient and transplant survival was better in AAV compared to nondiabetic patients [45]. Various single and multi-center cohort studies have evaluated the outcomes of renal transplantation in patients with AAV [8,11,12,19,22,46-51] and have shown that the rate of patient and transplant survival in AAV is equal and comparable to control nondiabetic patients. The five-year post-transplant patient survival ranges between 77% and 96%, and the five-year graft survival is between 60% and 100%. In addition, one study, in particular, compared the post-transplantation death hazard ratios across various GN subtypes and elucidated that while patients with IgA nephropathy (IgAN) had the lowest mortality rates, patients with IgAN or vasculitis had the lowest allograft failure rates [52].

Appropriateness of kidney transplant timing

Until recently, no clear data addressing the optimal timeframe for renal transplantation in AAV patients existed. However, in 2009, a study conducted by Little et al. demonstrated that patients who underwent renal transplantation prior to achieving 12 months of AAV disease remission had a higher risk of death in both univariate and multivariate analysis [12]. Ever since multiple society guidelines have endorsed the achievement of remission for one year prior to renal transplantation [53,54]. However, this only remains a recommendation since the causes of death were unrelated to recurrent vasculitis. In addition, current guidelines recommend against the delay of renal transplantation in patients who reach remission for the recommended time frame but remain ANCA-positive. Based on available studies, the serological status of ANCA-positivity appears to be unrelated to both, the rate of post-transplantation relapse and the rate of graft or patient survival [48].

Post-transplant recurrence of AAV

Currently, there is no prospective data assessing the likelihood of recurrent ANCA vasculitis after kidney transplantation. However, the recurrence frequency following transplantation has been assessed in several retrospective case series [12,48,50]. These have revealed a frequency of relapse around 15%-20%, although the frequency of recurrent pauci-immune necrotizing GN is only around 5%. One particular study observed AAV recurrence in only 2% of renal allografts [46]. However, compared to MPO-ANCA, PR3-ANCA patients are twice as likely to experience a relapse following renal transplant [8].

While current guidelines do not deter providers from offering renal transplantation to patients with ESKD on the premise of ANCA positivity alone, whether or not ANCA serological status plays a role in relapse remains a contentious topic. Nachman et al. did not elucidate an association between post-transplant relapse and ANCA positivity. However, the study carried out by Geetha et al. showed a marginal significance (p=0.05) between the ANCA serological status and increased risk of relapse following transplantation [8]. Another study showed that while there was no relationship between ANCA positivity and relapse, ANCA-positive recipients were more likely to develop vasculopathy-related complications in the graft. Based on the data, it is safe to conclude that the risk of relapse poses a grim possibility for patient outcomes. However, with the advent of modern post-transplant immunosuppressive therapy, relapse represents a nominal risk that is worthwhile taking.

The post-transplant therapeutic regimen to prevent disease recurrence and allograft rejection has also been studied. Observational studies have shown that the combination of mycophenolate mofetil, prednisone, and tacrolimus has decreased vasculitis relapse rates post-transplant to 5%-10% with very few recurrences in the allograft [8,50]. Therefore, with the aid of modern immunosuppressive agents, the risk of extra-renal flares and disease recurrence within the graft is minimal. In the event of a severe relapse, both cyclophosphamide and rituximab have proven effective in the induction of remission following renal transplantation [21,48]. Of note, both the rituximab in ANCA-AAV (RAVE) trial and rituximab versus cyclophosphamide in ANCA-AAV (RITUXVAS) trial showed that rituximab is non-inferior to cyclophosphamide in inducing remission of severe ANCA-AAV when combined with glucocorticoids. However, their patient profile consisted of either a new diagnosis or relapsing disease; it did not assess the post-transplant population [55,56].

Disease process in RA

The traditional viewpoint on the implication of the renal system as a direct consequence of RA is that it represents a rare occurrence. Renal impairment in the setting of RA is usually attributed to anti-rheumatic drug toxicity (e.g., NSAIDs), chronic inflammatory disease processes (e.g., Amyloidosis), or the emergence of an overlapping rheumatic disease [4,57,58]. In a retrospective cohort study conducted by Kochi et al., inflammatory states in the form of a persistently elevated CRP were coined as a significant risk for the development of CKD in RA patients [57]. In addition, the fact that the detriment of renal function in RA leads to increased morbidity and mortality has been widely reviewed [59,60]. For this reason, determining the long-term risk of deterioration of renal function in an otherwise uncomplicated and stable RA patient as a potential outcome for the donor, was of utmost importance.

It has not been until recent decades that the possibility of renal disease in otherwise uncomplicated RA patients has gained wider awareness among the medical community. Retrospective histopathological data suggest a close clinicopathological correlation between RA and renal function compromise. Studies suggest the direct effects of RA in renal function include the potential development of mesangial GN, amyloidosis, focal proliferative GN, MN, among others, with mesangial GN being the most frequent lesion [3-4,60]. In addition, the 20-year retrospective cumulative study conducted by Hickson et. al. showed that the incidence of reduced kidney function was higher in patients with RA compared with non-RA participants at various points of the disease process. Overall, it elucidated that the cumulative incidence for impaired renal function (defined as eGFR < 60) was 9% at baseline, 15% at 10 years, and 25% at 20 years [1,59].

As previously mentioned, medications commonly used in RA have been widely associated with renal impairment in this patient population. The use of NSAIDs, in particular, has been associated with interstitial nephritis, minimal change disease, and membranous GN [61]; it decreases renal blood flow and glomerular filtration by dilating the efferent arterioles [62,63]. Disease-modifying antirheumatic drugs (DMARDs) such as cyclosporine, d-penicillamine, and gold-containing compounds have often been linked to renal impairment [64,65]. Meanwhile, DMARDs, such as MTX, azathioprine, leflunomide, mycophenolate, and sulfasalazine, have been found to have a lesser effect on renal function [59]. Recipient age alone does not represent a contraindication to renal transplantation [65-67].

Outcomes of kidney donation in RA patients

Despite exhaustive research, scant data was unearthed regarding the topic of kidney donation among individuals with RA. Similarly, we were unable to find other studies involving outcomes for RA patients undergoing other organ donations. However, one comparative study conducted by Cheungpasitporn et. al. showed that six healthy kidney donors with RA had auspicious post-donation outcomes in parallel to 18 non-donor counterparts [5]. At a median follow-up of 5.9 and 8.2 years, there were no donor deaths or reported developments of ESKD. In addition, at the 5.9-year follow-up, no donors had experienced an RA flare. In contrast, 27% of non-donors with RA experienced flares at a median follow-up of 4.7 years. Even though the positive results of this study boded well for our patient, it is important to note that, unlike our (potential) donor, all six study subjects had reached clinical remission at the time of transplant and were considered to be otherwise healthy individuals.

Recommendations and decision-making

This unique clinical scenario was presented at our multidisciplinary kidney selection and donor advocate transplant meetings. As previously mentioned, multiple opposing viewpoints were conveyed. Even though our potential recipient had a complex medical history in the setting of atypical AAV requiring three years of renal replacement therapy, the decision for him to undergo renal transplantation was unanimous. The survival benefit of renal transplantation in AAV was first demonstrated in 1976 [13]. Modern immunosuppression has improved both patient and graft survival following kidney transplantation over the last few decades. It has been well known that patient survival is better with kidney transplants among patients with ESKD. In our potential recipient, the benefits of undergoing renal transplantation greatly outweighed the risks. At the time of pre-transplant assessment, he had no active infections, malignancy, substance abuse, uncontrolled psychiatric problems, documented treatment non-adherence, or signs of reversible kidney function. Also, our recipient had been on clinical remission with rituximab for longer than the 1-year stipulated period.

However, whether the kidney should stem from our 60-year-old female patient with RA, not in remission, remained a topic of contention. Drug-mediated toxicity was of little concern for our donor. Her medical management consisted of hydroxychloroquine and Tylenol. She had no record of ever using renally toxic DMARDs, such as cyclosporine, d-penicillamine, or gold-containing compounds. In addition, given her extensive gastrointestinal issues, she had never tried NSAIDs for pain control. However, as previously mentioned, she calms her pain flares with Tylenol, an acetaminophen-containing compound that has been found to cause acute papillary necrosis and progressive renal failure at elevated doses [68]. In addition, our donor was morbidly obese, had a past bariatric surgery history, and was a former smoker. Despite the auspicious data elucidated from the comparative study previously mentioned, our potential donor’s overall clinical picture was far more precarious than that of the six otherwise healthy RA donor subjects from the aforementioned study. Based on current guidelines, morbid obesity represents a relative contraindication beyond the Organ Procurement and Transplant Network (OPTN) requirements [69,70]. Meta-analysis studies have revealed that North American donors with higher BMIs at the point of donation carry a higher risk of 20-year post-donation ESKD compared to healthy non-donors [70-72]. In addition, a single-center retrospective study showed that for the local patient population in Hershey, Pennsylvania, obesity is a prominent risk factor associated with postoperative ICU admissions following noncardiac elective procedures [73]. For this reason, the decision to approve such a candidate was a complex and individualized decision that needed to balance the donor’s health profile with a clinically acceptable risk threshold.

## Conclusions

Our team felt that our potential recipient would undoubtedly benefit from renal transplantation. However, the potential donor faced too many unknowns in the advent of living kidney donation, tipping our risk assessment scale towards recommendation against the donation of her kidney. Fortunately, for our recipient, a second donor was identified, representing a more viable option than our patient with RA. Exploring the data that supported or refuted our original conundrum was imperative. After much deliberation, we concluded that even in the absence of a second donor, our best recommendation is to find an alternative means of transplantation for our recipient. Our recipient successfully underwent an unrelated living donor renal transplant in February 2021, and his repeat ANCA status test was nonreactive at the one-month post-transplant visit.
